# Prurigo Pigmentosa Associated with a Ketogenic Diet in a Romanian Patient: A Case Report

**DOI:** 10.3390/healthcare13030300

**Published:** 2025-02-02

**Authors:** Carmen Andrada Iliescu, Cristina Beiu, Tiberiu Tebeică, Liliana Gabriela Popa

**Affiliations:** 1 Clinic of Dermatology, Elias Emergency University Hospital, 011461 Bucharest, Romania; carmenandrada1997.iliescu@gmail.com (C.A.I.); liliana.popa@umfcd.ro (L.G.P.); 2 Department of Oncologic Dermatology, Elias Emergency University Hospital, Carol Davila University of Medicine and Pharmacy, 020021 Bucharest, Romania; 3 Department of Histopathology, Dr. Leventer Centre, 011216 Bucharest, Romania; tiberiutebeica@drleventercentre.com

**Keywords:** prurigo pigmentosa, Nagashima’s disease, ketogenic diet, reticulated pigmentation

## Abstract

**Background/Objectives:** Prurigo pigmentosa (PP) is a rare inflammatory dermatosis characterized by recurrent pruritic eruptions resolving with reticulated hyperpigmentation. Initially described in young Asian women, PP is increasingly reported worldwide, often linked to ketosis-inducing factors such as low-carbohydrate diets. This report documents the first case of PP in a Romanian patient and highlights the importance of recognizing the condition in diverse populations. **Methods:** We describe a 16-year-old girl with a six-month history of pruritic eruptions on the neck, trunk, shoulders, and thighs. A detailed clinical assessment was conducted, including dietary history, physical examination, laboratory evaluation of urinary ketones, and skin biopsy for histopathological analysis. **Results:** Clinical examination revealed erythematous papules and plaques with peripheral pustules and post-inflammatory hyperpigmentation. Substantial weight loss and elevated urinary ketone levels suggested dietary-induced ketosis from a strict low-carbohydrate diet. Histopathological findings confirmed the diagnosis of PP. The patient’s skin lesions resolved following carbohydrate reintroduction and doxycycline therapy. **Conclusions:** This case highlights the association between ketosis and PP, emphasizing the importance of thorough dietary history-taking and histopathological confirmation for accurate diagnosis. As ketogenic diets become more popular globally, heightened awareness of PP is essential to ensure timely diagnosis and effective management.

## 1. Introduction

Prurigo pigmentosa (PP) is an idiopathic inflammatory dermatosis first described by Nagashima in 1971 [1]. Although the majority of cases have been documented in young women of Asian descent, recent reports indicate its occurrence across various ethnicities worldwide [2,3]. Clinically, PP is characterized by recurrent pruritic eruptions, presenting as erythematous macules, urticarial papules, or papulovesicles that merge into a distinctive reticulated pattern, predominantly affecting the neck and trunk [3]. The lesions resolve rapidly, with most cases subsiding within weeks, leaving behind an asymptomatic reticulated hyperpigmentation [4]. Various endogenous factors, such as ketosis, diabetes mellitus, and anorexia nervosa, as well as exogenous factors, including clothing friction, sweating, and contact allergens, have been suggested as potential inciting agents for PP [5]. Standard treatments include oral antibiotics, particularly tetracyclines, macrolides, and dapsone, in conjunction with dietary adjustments [6,7].

To our knowledge, this is the first documented case of PP in a Romanian patient. This report explores both the clinical and histological features of this rare dermatosis and provides a thorough review of the literature.

## 2. Case Report

A 16-year-old Romanian girl presented to our Dermatology Department with a six-month history of recurrent pruritic, erythematous eruptions, primarily localized to the neck and trunk, with minor involvement of the shoulders and upper thighs. Her medical history included allergic rhinitis and dermographism, as well as a positive family history of pustular psoriasis.

**Figure 1:**
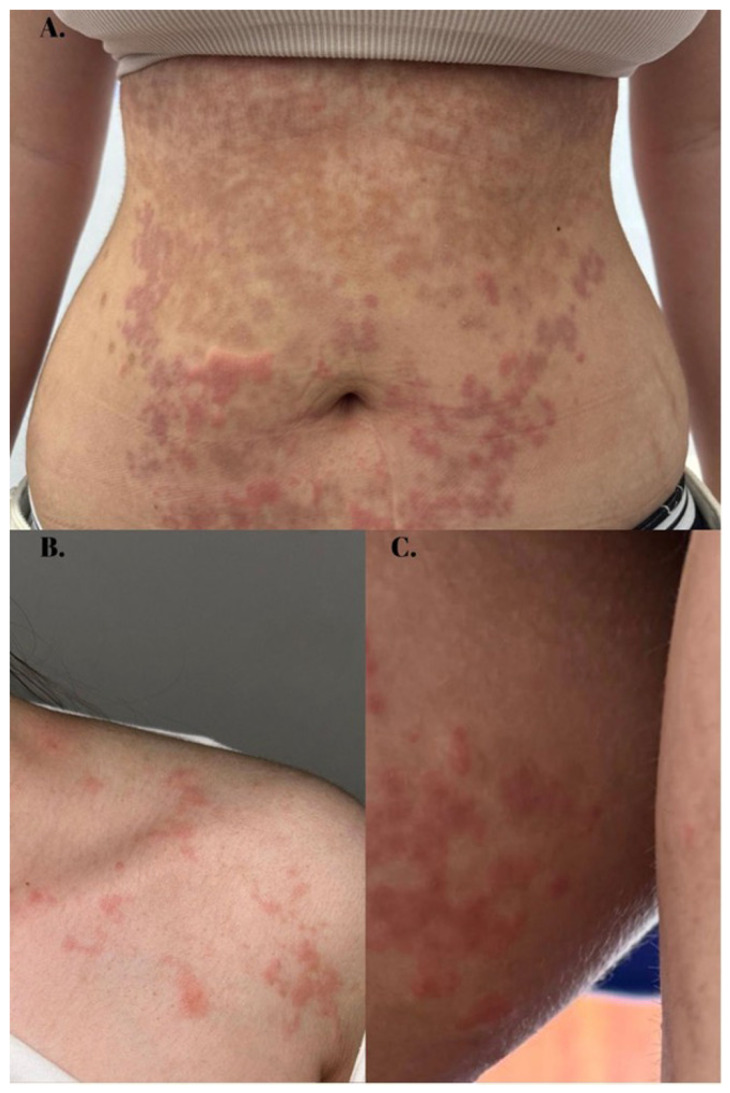
Clinical aspect of PP. (**A**) Pruritic erythematous urticarial papules and plaques with small peripheric non-follicular pustules on the abdomen; (**B**,**C**) residual netlike hyperpigmentation following inflammatory lesions resolution, with similar erythematous papules and plaques observed on the shoulder and thigh region.

On physical examination, erythematous–violaceous urticarial papules and plaques with small peripheral pustules were symmetrically distributed in an annular pattern across the neck, trunk, and upper thighs (Figure 1). Additionally, a reticular pattern of post-inflammatory hyperpigmentation was noted on the abdomen (Figure 1A). There was no involvement of hair, nails, or mucous membranes. The patient denied any history of sensitivity to foods or cosmetics and reported no drug exposure before the rash onset. Notably, she had been following a strict low-carbohydrate diet for several months before the eruption onset. During this period, the patient achieved successful weight loss without experiencing any adverse effects. Previous treatments with systemic corticosteroids, systemic and topical antifungals, and topical corticosteroids—prescribed in another clinic—proved ineffective.

Differential diagnoses included urticarial vasculitis, generalized pustular psoriasis, and annular pityriasis versicolor. Comprehensive metabolic and autoimmune evaluations, including complement levels (C3, C4), antinuclear antibody profile, Anti-SSA (Ro) and Anti-SSB (La) antibodies, IgE serum levels, and thyroid function tests, were within normal limits, except for elevated urinary ketone bodies (80 mg/dL), indicative of dietary-induced ketosis. Infectious screening, including testing for anti-*Borrelia* antibodies and *Helicobacter pylori*, yielded negative results. Hormonal fluctuations during the menstrual cycle were assessed and found to be unremarkable.

**Figure 2:**
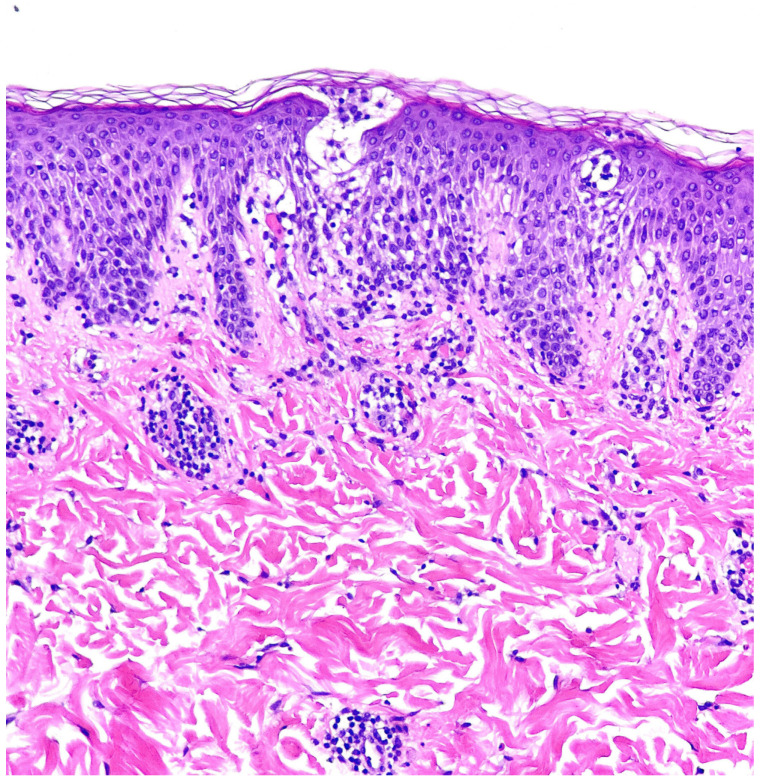
Histopathological aspect of lesions. Spongiosis with exocytosis of lymphocytes and sporadic neutrophils, necrotic keratinocytes, and a mixed perivascular inflammatory infiltrate in the dermis composed of lymphocytes, eosinophils, and histiocytes.

A punch biopsy was obtained from an active abdominal lesion to clarify the diagnosis. Histopathological analysis revealed a spongiotic epidermis with exocytosis of lymphocytes and occasional neutrophils, along with scattered necrotic keratinocytes (Figure 2). Additionally, a superficial perivascular inflammatory infiltrate, consisting of lymphocytes, eosinophils, and histiocytes, was observed in the dermis. Based on these clinicopathological findings and the patient’s dietary history, a diagnosis of PP was established.

Based on these clinicopathological findings and the patient’s dietary history, a diagnosis of PP was established, most probably in the context of a low-carbohydrate diet. Standard patch testing to assess potential contact allergens as triggers for the eruption was negative. No evidence of other potential exogenous triggers, such as mechanical friction, trauma, or sweating, was identified as exacerbating factors for the eruption.

**Figure 3:**
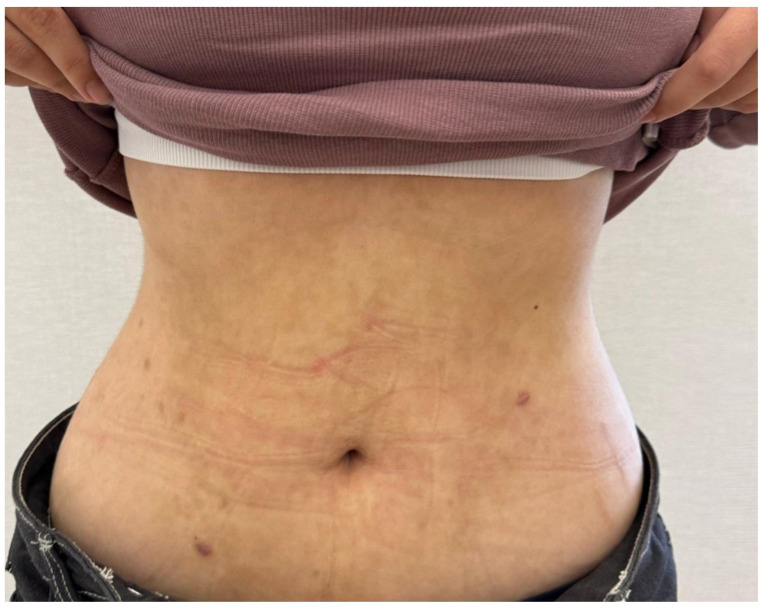
Dramatic improvement of PP after two months of treatment.

Given the association between PP and ketosis, the patient was advised to reintroduce carbohydrates into her diet. She was also initiated on doxycycline at a dosage of 100 mg/day. Within two months, the rash had completely resolved, with no subsequent recurrences and a gradual fading of the reticular pigmentation (Figure 3).

After the reintroduction of carbohydrates, and 100 mg of Doxycycline per day, the residual reticulated hyperpigmentation significantly faded, and no active lesions were visible on the abdomen.

Three months after completing her course of doxycycline, the patient has not experienced any recurrences, and her condition remains stable, maintained solely through dietary modifications.

## 3. Discussion

Prurigo pigmentosa, also known as “Nagashima disease” or “keto rash”, is a rare inflammatory skin condition marked by recurrent pruritic eruptions that heal with a reticulated pigmentation pattern [8]. While PP has mainly been documented in the Japanese population, recent case studies indicate a rising occurrence across various ethnic communities worldwide [9]. This trend suggests that limited clinical awareness outside Asia, rather than any genetic factors, might have contributed to underdiagnosis in these regions [8]. PP most frequently affects females in their second or third decade of life, with a reported female-to-male ratio of 2:1. Fewer cases have been noted in prepubescent children and older adults [10]. Our case of a 16-year-old Romanian girl aligns with reported demographic patterns in the literature, yet as the first documented case in a Romanian patient, it expands the known epidemiological range of PP beyond Asia into Eastern Europe. This underscores the need for increased clinical recognition of PP among healthcare professionals in diverse geographic areas, especially as low-carbohydrate dietary practices gain more global popularity.

Clinically, PP consists of cyclic eruptions of erythematous macules, urticarial papules or vesicles that may coalesce to form an arcuate or reticular pattern [9]. Bullous and pustular variants have also been noted [11,12]. The rash is mainly symmetrically distributed in the neck and chest area, although lesions in the axilla, pubic region, and buttocks may also be present [9]. Individual lesions typically persist for days and resolve with asymptomatic post-inflammatory hyperpigmentation in a net-like distribution. Recurrences are frequent in the course of the disease and might appear even years after the initial diagnosis [13]. Our patient exhibited clinical features similar to those described in the literature, with lesions in various stages of development noted at the time of presentation: urticarial papules, vesicles, pustules, and post-inflammatory hyperpigmentation.

Although the precise mechanism of PP has yet to be determined, several endogenous and exogenous factors have been associated with the disease (Table 1) [3]. Endogenous factors include atopic diathesis, Sjögren syndrome and infections with *Helicobacter pylori* or *Borrelia* spirochetes [5,14,15,16]. Hormonal influences, particularly estrogen, may also play a role in PP pathogenesis, as flare-ups have been reported during menstrual cycles and pregnancy [9]. Possible aggravating external factors include mechanical friction, sweating, physical trauma, and contact with allergens such as nickel and para-amino substances [4,17].

Recently, PP has been linked to various ketotic states, including poorly controlled diabetes mellitus, intermittent fasting, anorexia nervosa, a low-carbohydrate diet, and post-bariatric surgery [8,18]. The reduced blood glucose or insulin levels observed in these conditions can stimulate hepatic ketogenesis, resulting in elevated ketone body concentrations detectable in blood or urine [6]. These ketones may enter cells, disrupt intracellular processes, or accumulate around blood vessels, leading to a predominantly neutrophilic inflammatory response [19]. In our case, the onset of PP appeared to be temporally associated with the patient’s adherence to a ketogenic diet, as evidenced by elevated urinary ketone levels indicative of dietary-induced ketosis. The patient’s atopic diathesis may have played a contributory role; however, their allergic rhinitis had been well-controlled for years, dermographism was minimal, and no prior flares of atopic dermatitis had been reported. Comprehensive laboratory investigations excluded other endogenous causes, including a negative immunologic profile, negative infectious screening, and normal hormonal levels without clinical signs of hormonal imbalance. Exogenous causes were also ruled out through standard patch testing and a negative anamnesis for mechanical friction, trauma, or sweating as exacerbating factors for the eruption. As the ketogenic diet grows in popularity, especially among young people, recognizing this association is crucial for prompt diagnosis and intervention in similar cases.

While early reports described the histopathological findings of PP as nonspecific and lacking diagnostic significance, recent evidence indicates that PP displays some distinct, stage-specific histopathological characteristics [5]. In the early stages, consisting of erythematous macules and urticarial papules, a superficial perivascular inflammatory infiltrate predominantly composed of neutrophils is observed, accompanied by mild spongiosis and a few necrotic keratinocytes [13]. As lesions progress and fully develop, neutrophils are replaced by lymphocytes and eosinophils, which assume a more profound lichenoid distribution [3]. In the final resolution phase, dermal melanophages are present alongside a sparse lymphocytic infiltrate [2]. The histopathological observations in our case align with this proposed stage-specific progression.

Subsequently, recent publications have studied the immunohistochemical profile of PP, suggesting that myeloperoxidase and CD11 staining may reveal myeloid precursor cells within the inflammatory infiltrate. This finding indicates a potential role for immature myeloid cells in PP pathogenesis, providing an additional diagnostic tool, especially in cases with a paucity of mature neutrophils [20].

The differential diagnosis for inflammatory lesions in PP commonly includes acute lupus erythematosus, dermatitis herpetiformis, and linear IgA dermatosis [21]. However, in cases where patients present with residual hyperpigmentation, alternative diagnoses should be considered, including ashy dermatosis, Gougerot-Carteaud syndrome (confluent and reticulated papillomatosis), erythema dyschromicum perstans, and pigmented contact dermatitis [22]. However, in our case, these conditions were neither strongly suspected based on anamnesis nor supported by histopathological examination, which was negative for features consistent with these diagnoses. Furthermore, our report expanded the differential diagnosis to include several conditions not commonly addressed in the literature on PP. Annular pityriasis versicolor was considered due to the distribution and annular configuration of the lesions, while urticarial vasculitis emerged as a possibility given the presence of urticarial papules with residual hyperpigmentation. Additionally, pustular psoriasis was taken into account due to the patient’s family history and the presence of non-follicular pustules. As a consequence, this case underscores the necessity of considering an expanded range of differential diagnoses, as the clinical presentation here diverged from the classical differentials described in the literature.

Various therapeutic options exist for managing PP with oral antibiotics, particularly tetracyclines (doxycycline and minocycline) and sulfonamides (dapsone), being the most favored choices [23]. These antibiotics are supported by their anti-inflammatory properties, notably by inhibiting proinflammatory cytokine expression, neutrophil function, and chemotaxis [5,24]. Despite comparable efficacy, doxycycline at 100 mg daily is preferred over minocycline and dapsone due to its superior safety profile [25]. Other drugs reported to be beneficial include isotretinoin and macrolide antibiotics [7,11,26]. Antihistamines and corticosteroids (both topical and systemic) have limited roles in the treatment of PP [27]. In addition to pharmacological interventions, reintroducing a balanced diet, particularly in patients with a history of restrictive dieting, and addressing any underlying hyperglycemia or dehydration may facilitate disease resolution as adjuvant measures [19,28]. In our case, the patient demonstrated a significant response to combined therapy with doxycycline and dietary adjustments. The patient was also counseled on the risk of recurrence associated with future fasting, dieting, or other ketotic states.

Our case is limited by the short-term follow-up period of three months, which, although showing no recurrence of lesions, provides only preliminary evidence regarding the efficacy of dietary modifications in preventing future episodes. Longer-term monitoring is necessary to determine whether dietary changes alone can sustain resolution. Additionally, as a single case study, the findings are not generalizable and require validation through larger cohort studies and controlled nutritional interventions. Future research is needed to establish a more definitive causal link between ketogenic diets and prurigo pigmentosa and to develop standardized management protocols for diagnosis and treatment.

## 4. Conclusions

PP remains an underrecognized inflammatory dermatosis outside Asia, despite its growing global prevalence due to lifestyle changes, including ketogenic dieting. This case underscores the necessity for heightened clinical awareness of PP in diverse ethnic groups and geographic locations. Our report demonstrates the pivotal role of dietary history in the diagnostic process, particularly in identifying ketosis as a potential trigger. Furthermore, this case reinforces the effectiveness of combining dietary modifications with anti-inflammatory treatments such as doxycycline. As the adoption of low-carbohydrate diets continues to rise, timely recognition and intervention will be essential to manage and prevent recurrences of PP effectively.

## Figures and Tables

**Table 1:** Endogenous and exogenous factors associated with prurigo pigmentosa (PP).

Endogenous Factors	Exogenous Factors
Atopic diathesis	Mechanical friction
Sjögren syndrome	Sweating
Infections (*Helicobacter pylori* and *Borrelia* spirochetes)	Physical trauma
Hormonal changes (pregnancy and the menstrual cycle)	Contact allergens (nickel, para-amino substances, etc.)
Ketotic states (diabetes mellitus, intermittent fasting, anorexia nervosa, a low-carbohydrate diet, and post-bariatric surgery)	

## Data Availability

Data are contained within the article.
